# Global change feed-back inhibits cyanobacterial photosynthesis

**DOI:** 10.1038/srep14514

**Published:** 2015-09-29

**Authors:** E. Walter Helbling, Anastazia T. Banaszak, Virginia E. Villafañe

**Affiliations:** 1 Estación de Fotobiología Playa Unión, Casilla de Correos 15, (9103) Rawson, Chubut, Argentina; 2 Consejo Nacional de Investigaciones Científicas y Técnicas (CONICET), Argentina; 3 Unidad Académica de Sistemas Arrecifales, Puerto Morelos, Instituto de Ciencias del Mar y Limnología, Universidad Nacional Autónoma de México, México

## Abstract

Cyanobacteria are an important component of aquatic ecosystems, with a proliferation of massive cyanobacterial blooms predicted worldwide under increasing warming conditions. In addition to temperature, other global change related variables, such as water column stratification, increases in dissolved organic matter (DOM) discharge into freshwater systems and greater wind stress (i.e., more opaque and mixed upper water column/epilimnion) might also affect the responses of cyanobacteria. However, the combined effects of these variables on cyanobacterial photosynthesis remain virtually unknown. Here we present evidence that this combination of global-change conditions results in a feed-back mechanism by which, fluctuations in solar ultraviolet radiation (UVR, 280–400 nm) due to vertical mixing within the epilimnion act synergistically with increased DOM to impair cyanobacterial photosynthesis as the water column progressively darkens. The main consequence of such a feed-back response is that these organisms will not develop large blooms in areas of latitudes higher than 30°, in both the Northern and Southern Hemispheres, where DOM inputs and surface wind stress are increasing.

Cyanobacteria are ubiquitous in aquatic systems^1^ and account for a great share of primary productivity by moving carbon dioxide and nitrogen from the atmosphere into the water column^2^. In temperate latitudes, toxic cyanobacterial blooms often occur in eutrophic ecosystems favoured by the development of a stable thermocline during warm months^3^,^4^. Moreover, increased eutrophication due to nutrient loading into freshwater systems acts synergistically with a warmer environment, promoting the dominance of cyanobacteria^5^,^6^. A proliferation of massive cyanobacterial blooms was predicted^3^ because their temperature optimum for photosynthesis and growth is higher than that for eukaryotic algae^7^, thus creating a competitive advantage under warming conditions. This creates serious consequences not only for the environment, e.g., by inducing mass mortality of fish and birds, but also for humans by altering the conditions of lakes and reservoirs^3^, many of which are used as sources of drinking water.

Global change, however, includes modifications in other variables such as precipitation and wind stress, which in turn, condition the amount of organic and inorganic material reaching a particular water body. The resulting interactions between solar radiation and other biotic or abiotic factors may have positive and negative feedbacks that have not been previously considered^8^. For example, increased surface water temperature due to global change not only intensifies the strength of the thermocline, but also will cause shoaling, thus reducing the depth of the upper mixed layer/epilimnion, further increasing the exposure of planktonic cells to solar radiation^2^,^9^,^10^. In contrast, increases in dissolved organic matter (DOM) discharge into freshwater systems^11^,^12^ reduces water transparency, which has been shown to negatively affect human health, because waterborne human pathogens normally killed by exposure to solar UVR^13^ might be spared under the reduced solar exposure^14^. On the other hand, increased opaqueness of the water column induces low-light acclimation in photosynthetic cells such that they have a greater susceptibility to solar UVR damage once they reach the surface of the water^15^. This differential acclimation has also been observed in other environments when comparing phytoplankton responses in a transect from opaque coastal to clear oceanic waters^16^, with higher amounts of photoprotective compounds in communities acclimated to clear oceanic conditions.

Previous studies^17^,^18^,^19^ have recognized that enhanced vertical mixing will play a significant role in controlling cyanobacterial blooms. However, experimentation assessing the role of global change variables in conjunction with vertical mixing is scarce^15^,^20^. Thus, we conducted experiments, using five cyanobacterial species as model organisms, to test the impact of global change related variables, including vertical mixing, on their photosynthesis. The photosynthetic responses of these cyanobacteria was then compared with the responses of natural phytoplankton communities from five different lakes where other taxonomic (i.e, Chlorophyceae, Bacillariophyceae, and Chrysophyceae) and competing groups were dominant.

## Results and Discussion

**Figure 1:**
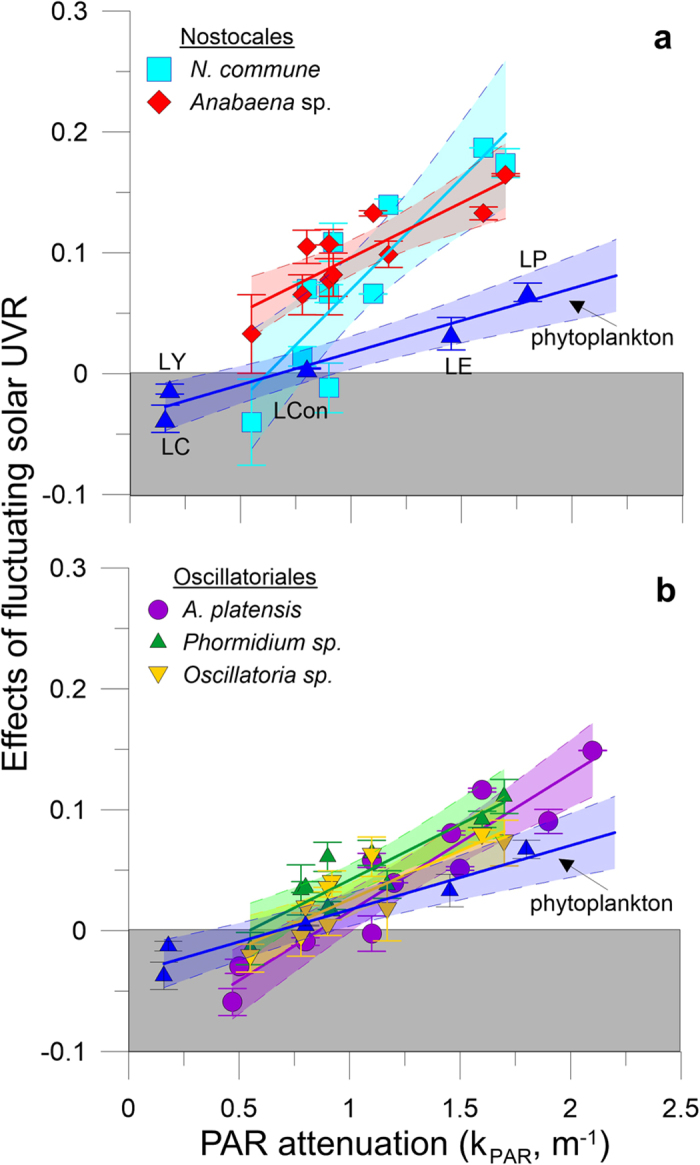
Impact of solar UVR on photosynthetic carbon assimilation (%) weighted by UV irradiance and depth of mixing for five cyanobacterial species and natural phytoplankton communities from five lakes as a function of water column transparency. The effect is expressed as the difference in the UVR inhibition of the samples under fluctuating radiation when moving within the epilimnion and the integrated UVR inhibition in the epilimnion calculated from static samples under three fixed irradiances. The penetration of solar radiation into the water column is expressed as the attenuation coefficient for PAR (k_PAR_, m^−1^). (**a**) Nostocales: *Nostoc commune* (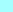), *Anabaena* sp. (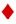). (**b**) Oscillatoriales: *Arthrospira platensis* (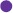), *Phormidium* sp. (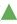), and *Oscillatoria* sp. (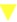). Each symbol represents one independent experiment (mean ± standard deviation; n = 3). The shaded areas in color represent the 95% confidence limits for the regression lines. The gray areas (i.e., effects < 0) indicate lower photosynthetic inhibition in samples incubated under fluctuating irradiance as compared to samples under constant irradiance. In both panels the responses of natural phytoplankton communities were added for comparison with those of cyanobacteria. In the figure, the data from the five different lakes are identified (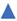), in addition to the slope and 95% confidence limits. LC, Lake La Caldera, dominated by Chrysophyceae; LY, Lake Las Yeguas, dominated by Bacillariophyceae; LCon, Lake La Conceja, dominated by Bacillariophyceae; LE, Lake Enol, dominated by Chlorophyceae; and LP, Lake Pipino, dominated by Chlorophyceae. For simplicity these labels are only shown in panel (**a)**.

In our experiments, we tested whether the combination of increased solar UVR (as a result of a shallower epilimnion and more frequent circulation near the surface), fluctuating irradiances (as a result of stronger vertical mixing due to increased wind stress^21^,^22^) and attenuation of solar radiation in the water column (as a result of inputs of dissolved and particulate material in the water column from land use, rain, wind resuspension, etc.) resulted in antagonistic or synergistic impacts favouring or impairing cyanobacterial photosynthesis. We found significant inhibition of solar UVR on photosynthesis of all species tested (n > 30, P < 0.0066), with reduced carbon incorporation by 15% to 50% as compared to samples exposed only to PAR. The inhibition, however, differed between species, radiation quality, and transparency of the water column in both static (unmixed) and fluctuating (mixed) regimes. Under relatively clear water column conditions (i.e., low PAR attenuation, k_PAR _< 0.8 m^−1^), samples moving within the upper mixed layer (i.e., above the thermocline) and thus exposed to fluctuating solar irradiance had lower photosynthetic inhibition than samples receiving constant irradiances at fixed depths (negative values in the shaded gray area in Fig. 1). However, this pattern of photosynthetic inhibition completely reversed with increasing attenuation in the water column (k_PAR _> 0.8 m^−1^), with fluctuating UVR causing greater inhibition as the water column darkened (Fig. 1). *N. commune* (Fig. 1A) suffered greater inhibition with the increasing darkness of the water column (slope of the linear regression fit of 0.184; n = 30, R^2 ^= 0.74, P = 0.00084) as compared to the other species (slopes of the fits ranged from 0.081 to 0.114; n = 30, R^2 ^> 0.7, P < 0.0014). Nevertheless, all species showed a similar response (i.e., stronger photosynthetic inhibition) under fluctuating radiation as the attenuation of solar radiation increased in the water column, hinting that similar inhibition impacts were caused by the interaction of the variables tested here. Natural communities of phytoplankton (Fig. 1) had a similar response to the cyanobacteria, with increasing photosynthetic inhibition in fluctuating samples (as compared to the static ones) as the water column attenuation increased due to higher DOM. However, the photosynthetic inhibition in these natural communities was lower than that observed in the cyanobacteria under similar attenuation values. However, for water columns with PAR attenuation coefficients below 1 m^−1^ and 1.2 m^−1^, for Nostocales (Fig. 1A) and Oscillatoriales (Fig. 1B), respectively, there were no differences in the relative inhibition as compared to the natural phytoplankton. Nevertheless, the slope of the linear fit in these phytoplankton samples (i.e., 0.05; n = 15, R^2 ^= 0.9, P = 0.0068) was lower than for any of those determined for the cyanobacteria, indicating that the natural phytoplankton communities were less sensitive to the combined impact of fluctuating UVR, with increasing darkness in the water column.

**Figure 2:**
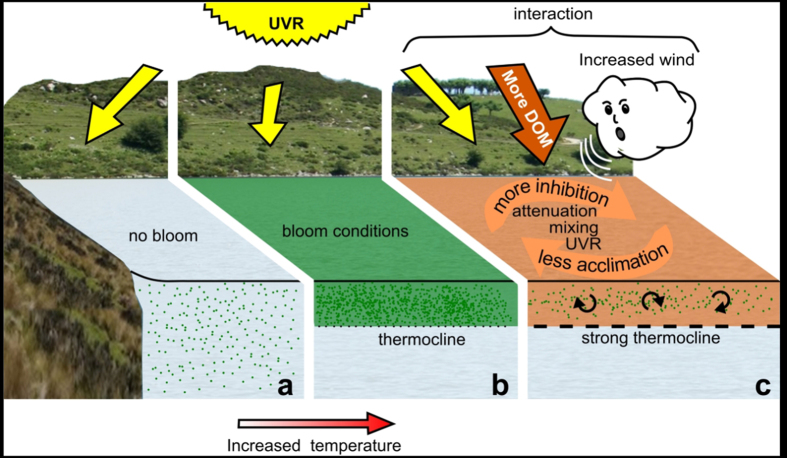
Conceptual model of (**a**) a turbulent scenario (deep mixing), (**b**) Predicted scenario of static conditions (no mixing) or (**c**) fast mixing within a shallow epilimnion under projected global change conditions. In (**a**) and (**c**) there are reduced chances of a bloom event because in (**a**) wind prevents the development of a thermocline whereas in (**c**) the synergism between faster circulation, a turbid water column and fluctuating solar UVR, inhibits cyanobacterial photosynthesis and growth. Under an intermediate scenario (**b**) cyanobacterial blooms are expected to be more common in low or no mixed water conditions with a distinct thermocline. The photograph was taken by EWH and the figure was drawn by EWH.

In a turbulent and deeply mixed water column (Fig. 2A), cyanobacterial blooms cannot develop because mixing favours phytoplankton over buoyant cyanobacteria^19^,^17^. A rise in temperature will create a stratification of the water column^2^ resulting in a shallower epilimnion that will revert the competition to favour cyanobacterial blooms^3^ (Fig. 2B). Under this scenario, photochemical degradation of DOM^23^ will be enhanced under increased stratification because solar UVR exposure is greater. This process, however, is not strong enough to cope with the greater inputs of DOM that have been documented as a result of various factors acting alone or in combination, including changes in land-use patterns^24^, in soil and water acidity due to a decrease in sulfur deposition^11^,^12^,^25^, in nitrogen deposition^26^, and in precipitation^27^,^28^, as well as due to climate change^29^,^30^. For example, ca. 90% increase in dissolved organic carbon (DOC), one of the main components of DOM, has been reported in various lakes over the last two decades^11^. In addition to increasing stratification of the water column and more DOM, increases in wind stress of 7 to 27% are predicted for temperate areas^31^,^32^. The balance between wind input energy and strength of the thermocline will result in either a turbulent epilimnion leading to strong circulation of cells in a turbid water column (i.e., when wind force is not enough to erode the thermocline, Fig. 2C), or a deepening of the epilimnion (i.e., when wind force erodes the thermocline and mixes the water column, Fig. 2A).

Both of these extreme scenarios will bring about a decrease in cyanobacterial bloom formation. In the first case, a synergistic effect between the faster circulation of the cells with higher DOM concentration and fluctuating solar UVR will negatively affect photosynthesis and growth (Figs 1 and 2). In the case of a deeper epilimnion, the combination of slower circulation due to deeper mixing places the cells at a competitive disadvantage as proposed in Fig. 2 and in other studies^17^,^18^. Such turbulence and circulation^33^ will not only maintain cyanobacterial cells moving within the epilimnion but also will maintain phytoplankton species (such as diatoms) and particulate material from continental run-off in suspension, favouring turbidity within the epilimnion^18^. Also, inorganic and organic particles may remain longer within the epilimnion delaying a transition to clearer waters. Our short-term experiments suggest that the responses of the five cyanobacterial species to cope with UVR are slower than the environmental changes imposed by our fluctuating radiation setup. Under increased turbidity, acclimation processes appear to take longer, thus explaining the differential inhibition observed. Our results shed new light on the understanding of cyanobacterial photosynthetic response to global change variables and on the feed-back mechanisms involved in the development of cyanobacterial blooms. Our data show that the underlying interaction involved between the tested variables changed from antagonistic in relatively clear waters to synergistic with increasing opaqueness of the water column.

Greater fluctuations in irradiances due to vertical mixing and attenuation of solar radiation through the water column might help to counteract the expansion of cyanobacterial blooms, as their photosynthetic performance is shown here to be severely affected under such conditions. It has been reported^34^ that during one of the hottest summers in Europe (2003), intermittent artificial mixing of lake Nieuwe Meer failed to control a bloom of the harmful cyanobacterium *Microcystis*. However, while *Microcystis* numbers were low during the mixing part of the experiment, as soon as mixing was turned off a steep increase in cell numbers was recorded. Therefore our interpretation of these data is that mixing did in fact control the bloom. This previous field information, together with our experimental model of inhibition of cyanobacterial photosynthesis under future global change conditions will help to design water management strategies in low-wind environments where cyanobacterial blooms normally occur.

## Methods

Five cyanobacterial species were used as model organisms: Two species belong to the order Nostocales, *Nostoc commune* and *Anabaena* sp., and three, *Arthrospira platensis*, *Phormidium* sp. and *Oscillatoria* sp. to the order Oscillatoriales. Except for *A. platensis*, all genera have toxic representative species^35^,^36^,^37^ although in the particular case of the strains used in our study, toxicity was not tested. A total of 51 experiments were performed: 11 experiments with *A. platensis* and 10 experiments with each of the other four species. In order to compare the responses of the cyanobacterial species with those of natural phytoplankton communities, we used a subset of data from mixing experiments conducted previously in different lakes. The subset of experiments presented here are part of those published in Helbling *et al.*^15^, which used the same mixing set up for experimentation. This subset of data originated after the selection of experiments in which the irradiance received by the cells and temperature of the water column were as close as possible to the conditions that we imposed during the cyanobacterial experiments presented here. The data were obtained from the following lakes: LC, La Caldera (37˚ 03′ N, 3˚ 19′ W); LY, Las Yeguas (37˚ 02′ N, 3˚ 22′ W); LCon, La Conceja (38˚ 55′ N, 2˚ 47′ W); LE, Enol (43˚ 16′ N, 4˚ 59′ W); and LP, Pipino (23˚ 26′ N, 116˚ 42′ E).

### Experimental set up

To assess the combined effects of vertical mixing, solar ultraviolet radiation (UVR) and increasing attenuation of the water column on carbon incorporation, monospecific cultures in exponential growth of the five species of cyanonobacteria were placed into 20 ml quartz tubes, inoculated with 0.185 MBq of radiocarbon^38^ and exposed to solar radiation for two hours at local noon inside a water bath (3 m diameter, 1.4 m depth) for temperature control. Two radiation conditions were implemented: one set of samples, incubated in uncovered quartz tubes received ambient solar radiation (PAR + UV − A + UV − B, >280 nm, referred to as the PAB treatment) and another set of samples received only PAR because the tubes were covered with UV Opak 395 filter (Ultraphan, Digefra, referred to as the P treatment, >400 nm).

The tubes (3 clear and 1 dark per radiation treatment) were distributed in trays, with three of them placed at the surface, mid depth and bottom of the simulated epilimnion, respectively (static samples). The other tray (fluctuating samples) was displaced vertically from the surface to the bottom of the simulated epilimnion by a motorized, mixing simulator. This device imposes a constant velocity (10 cm min^−1^) to the tray, which results in an up-and-down trajectory of the PAB and P treatments tubes in the water column^15^. Water samples for the natural communities were treated in the same way as the cyanobacterial species.

Water column transparency was manipulated by adding DOM and particulate material collected from the local estuary run-off, such that the whole epilimnion was progressively darkened to represent different conditions of solar radiation penetration into the water column. To accomplish this, water with a high amounts of DOM and particulate material was collected from the river side of the Chubut River estuary (salinity < 0.6), and increasing volumes of river water was added to our incubation tank in order to progressively darken the water column. For each experiment/addition of high DOM water, we used a high resolution spectroradiometer (Ocean Optics HR 2000CG-UV-NIR) to calculate the attenuation of UVR and PAR in the water column, prior to initiating the experiment. Variations in DOM were estimated by absorption at 320 nm^39^.

### Analysis and measurements

Incident solar radiation at the water surface was monitored constantly with a broad-band ELDONET radiometer, whereas its penetration through the water column was measured using a spectroradiometer (Ocean Optics HR 2000CG-UV-NIR).

Carbon incorporation: After 2 h of exposure to solar radiation, the inoculated samples were immediately filtered under low pressure (<100 mmHg), through 0.7 μm Whatman GF/F filters (25 mm diameter). The filters were placed in 20-mL scintillation vials, and inorganic carbon was removed by exposing the filters to HCl fumes for 24 h. After acidification, scintillation cocktail (Ecoscint A) was added and the samples counted using a liquid scintillation counter^38^.

Chlorophyll-*a*: At the beginning of each experiment 50 mL of culture was filtered onto Whatman GF/F filters (25 mm diameter); the filters were placed in centrifuge tubes (15 mL) with 5 mL of absolute methanol and measured by fluorometric techniques^40^.

### Data treatment, calculations and statistics

The inhibition of photosynthesis due to UVR (UVR_inh_) was calculated as:


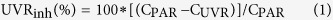


where C_PAR_, and C_UVR_ represent the carbon fixed in samples under the PAR only, and PAR + UV − A + UV − B treatments, respectively.

In the case of static samples, carbon fixation was integrated over depth for the simulated epilimnion and the inhibition of UVR calculated as mentioned above.

In all experiments, triplicates were used for all conditions, and the combined effects of vertical mixing, solar ultraviolet radiation (UVR) and attenuation of the water column were assessed using a 3-way. Analysis of Variance (ANOVA) in combination with a post hoc test (Tukey´s HSD).

## Additional Information

**How to cite this article**: Helbling, E. Walter *et al.* Global change feed-back inhibits cyanobacterial photosynthesis. *Sci. Rep.* **5**, 14514; doi: 10.1038/srep14514 (2015).
